# Supplementary material for: Computer-based quantitative image texture analysis using multi-collinearity diagnosis in chest X-ray images
Source: PLoS One. 2025 Apr 14;20(4):e0320706. doi: 10.1371/journal.pone.0320706 (PMC11996224; doi:10.1371/journal.pone.0320706)

S1 Fig. Confusion Matrices without the tuning weight parameter during training for Class 0 (normal), Class 1 (COVID-19), Class 2 (viral pneumonia), and Class 3 (lung opacity). (**a**) Observations, (**b**) TPR and FNR responses, (**c**) PPV and FDR responses.

(a) Confusion matrix displaying classification counts for all observations

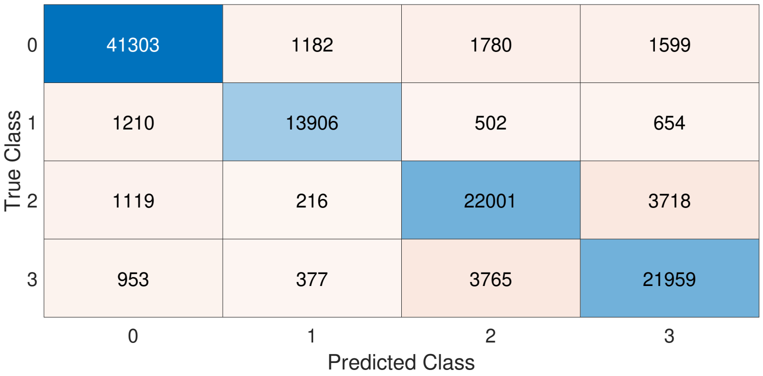

(b) Matrix displaying True Positive Rates (TPR) and False Negative Rates (FNR)

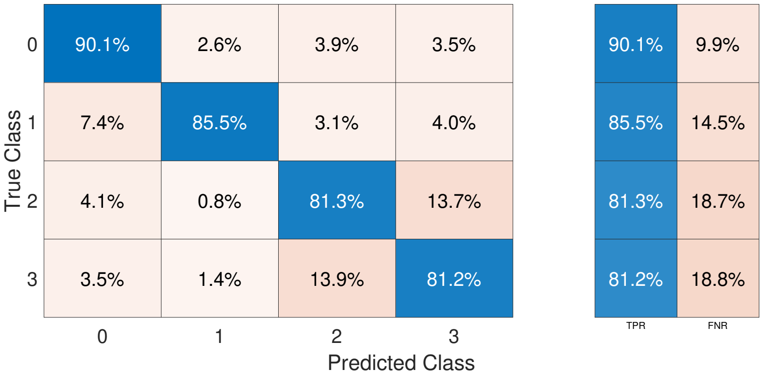

(c) Matrix showing Positive Predictive Value (PPV) and False Discovery Rate (FDR)

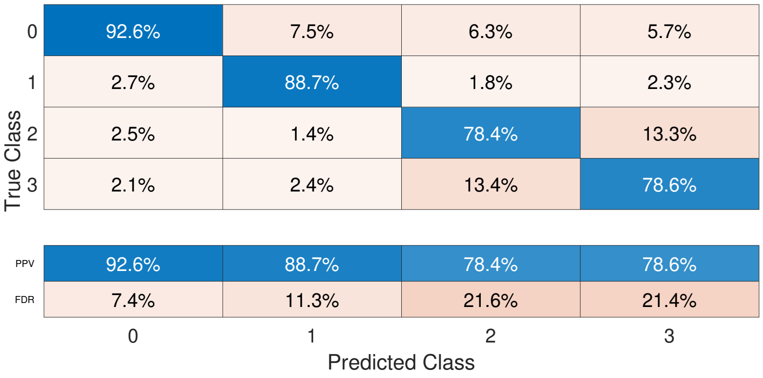

Supplement: S1 Fig — (PDF) [file pone.0320706.s001.pdf]
