# Supplementary material for: Computer-based quantitative image texture analysis using multi-collinearity diagnosis in chest X-ray images
Source: PLoS One. 2025 Apr 14;20(4):e0320706. doi: 10.1371/journal.pone.0320706 (PMC11996224; doi:10.1371/journal.pone.0320706)

S3 Fig. Receiver Operating Characteristic (ROC) and Area Under the Curve (AUC) of the features without the tuning weight ω: Class 0 (normal, blue dots), Class 1 (COVID-19, red dots), Class 2 (viral pneumonia, purple dots), and Class 3 (lung opacity, yellow dots).

(a) ROC for learning stage

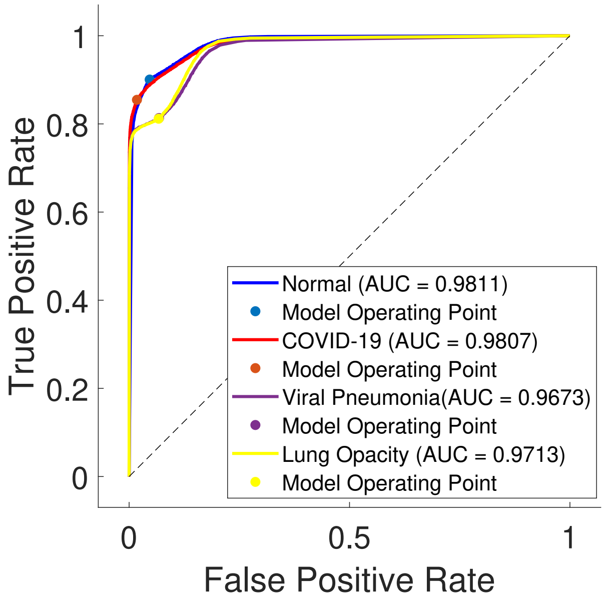

(b) ROC for testing stage

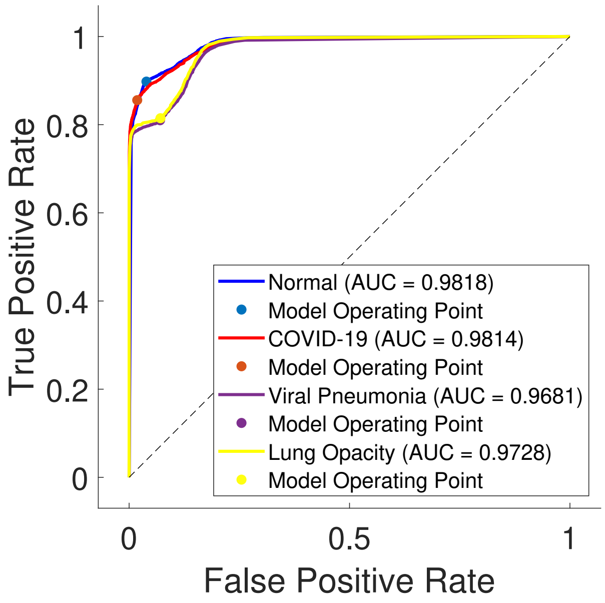

Supplement: S3 Fig — (PDF) [file pone.0320706.s003.pdf]
