# Supplementary material for: Computer-based quantitative image texture analysis using multi-collinearity diagnosis in chest X-ray images
Source: PLoS One. 2025 Apr 14;20(4):e0320706. doi: 10.1371/journal.pone.0320706 (PMC11996224; doi:10.1371/journal.pone.0320706)
Supplement: Attachment — Submitted filename: PONE-D-24-57634 Response to Reviewers.pdf [file pone.0320706.s006.pdf]

February 8, 2025

Response to the editor and reviewers of PONE-D-24-57634.
Computer-based quantitative image texture analysis using multi-collinearity diagnosis in chest X-ray images.
PLOS ONE
Antonio Quintero-Rincón, Ricardo Di-Pasquale, Karina Quintero-Rodríguez, Hadj Batatia.

We thank the editor and the three reviewers for their careful and thoughtful examination of our paper. We appreciate the time and effort they have put into their reviews. Below, we provide answers to all comments. The main changes made to the manuscript are shown in red. We also proofread the whole text and made many changes (not shown in red) to correct some typos and improve the writing style for better readability.

Response to Reviewer #1

Reviewer: *Authors have purposed a method to classify viral infection based chest X-ray images based on the singular values and the condition indices to characterize image texture using an SVD decomposition. It is noteworthy that the proposed method has low computational cost with imbalanced data as compared to other methods and good performance as measured by TPR, FNR, FDR, PPV, AUC, etc. With the emerging use of computer based quantitative image texture analysis for comprehensive and reproducible image analysis, this manuscripts adds value to the research community. I recommend to publish this manuscript in the current status.*

Authors: We thank the reviewer for their positive feedback and encouraging comments.

Response to Reviewer #2

Reviewer: *The authors introduces a novel method for automatic classification of chest X-ray images, aiming to differentiate between four categories: normal, COVID-19, viral pneumonia, and lung opacity. The paper proposes the use of singular values and conditional indices, extracted through SVD, as unique features to characterize tissue texture in chest X-rays. Traditionally, these parameters are used to diagnose multi-collinearity in regression models, but here they are adapted for texture analysis. To account for variability in X-ray attenuation caused by pathological changes, a tuning weight parameter, ω, is introduced. This parameter is derived using the coefficient of variation from the minimum covariance determinant of variance-decomposition proportions. The extracted features, weighted by ω are used in an ensemble bagged trees classification model. This approach addresses class imbalance effectively. In the results without weight tuning, conditional indices are shown to be the more significant predictors for classification. When weight tuning is included, singular values gain importance, and the overall separation between classes improves, particularly for COVID-19. The proposed method demonstrates*

high accuracy and computational efficiency, making it a promising tool for automated diagnosis of respiratory conditions in chest X-rays, specifically between COVID-19 and viral pneumonia, which can be hard to distinguish in chest X-rays.

Although I was able to follow the manuscript, it might need the help of a proofreading service to meet publication standards. Some of them are enumerated below, but my list is not exhaustive.

Reviewer: *Remark 2.1)*
Multiple instances of figure and figure references out of order throughout paper. Figures should be numbered consecutively in the order they are mentioned in the text. Move references to figures to match order of figure placement in body of paper, or move placement and order of figures to match reference order.

Authors: We recognise the confusing reference to Figure 3 in line 144 and apologize for the confusion. We have now moved the reference to line 194, where we explain the proposed method. All other figures have been reordered according to their references in the text. We have also made more explicit captions for all figures.

Reviewer: *Remark 2.2)*
Are Figures 10-14 part of the S1 appendix or part of main body? Supplementary figures are referred to as "Supporting Information" and should be numbered with an "S" prefix followed by a numeral (e.g., S1 Fig, S2 Fig). If Figure 10-14 are Supporting Information, update the caption and figure numbering appropriately and this would take care of several out of order figure references.

Authors: We thank the reviewer for this remark and apologize for the confusion. Figures 10-14 have now been captioned correctly in the "Supporting Information Section", with references "S1 Fig", "S2 Fig", and "S3 Fig" in lines 410 to 416; and with references "S4 Fig", "S5 Fig" in lines 425 to 428.

Reviewer: *Remark 2.3)*
Figure 12 is not referenced in paper at all, nor discussed in results.

Authors: We thank the reviewer for this remark and apologise for the confusion. Figure 12 is renamed Figure "S3" in the revised manuscript, and the associated results are now discussed in lines 412 to 416.

Reviewer: *Remark 2.4)*
Having the S1 Appendix figure occur in the middle of the references makes it difficult to read the references and papers.

Authors: We thank the reviewer for this remark. The appendix and the numbering of its figures are now correctly ordered. References are included in the right places in the text to provide explanations.

Reviewer: *Remark 2.5)*
Figure 5 color palette and symbol/line choice does not make the data easy to read and interpret. I would suggest to update the color palette for the data set to match Figures 6, 8, 9 for consistency. In addition, use different symbols/lines for data vs fit to make it easier to read the data. The color palette and use of solid lines for every data set made it hard to interpret the results.

Authors: We apologize for the inconvenience of using the inappropriate colour palette and line style and thank the reviewer for this valuable remark. We have now split the figure into four subplots, one for each class, to improve readability, preserving the same palette colours for each class; see Fig 5 (Figure 5 in the revised manuscript).

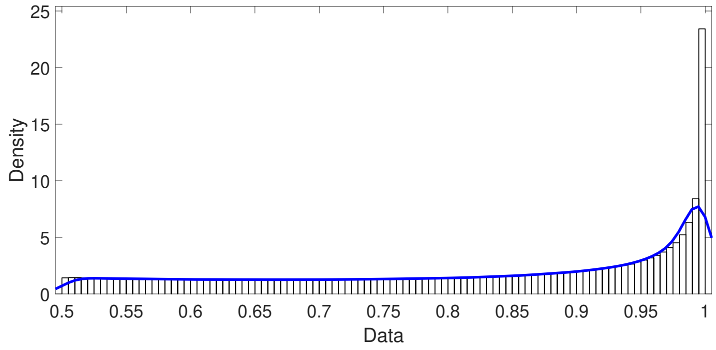

(a) Normal

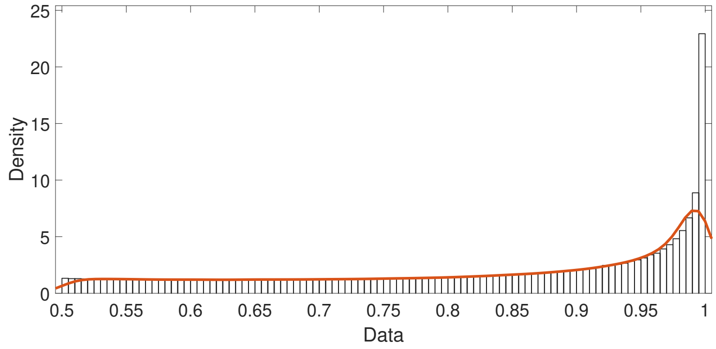

(b) COVID-19

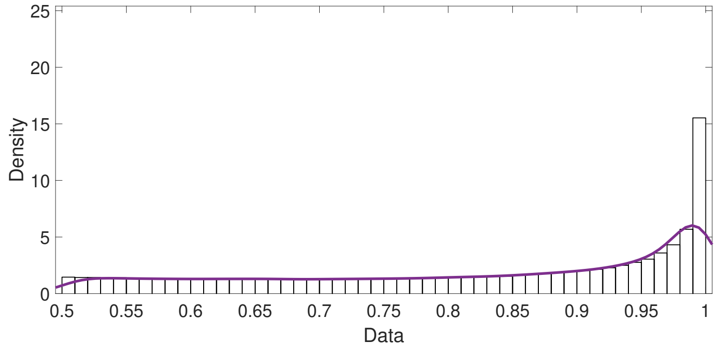

(c) Viral Pneumonia

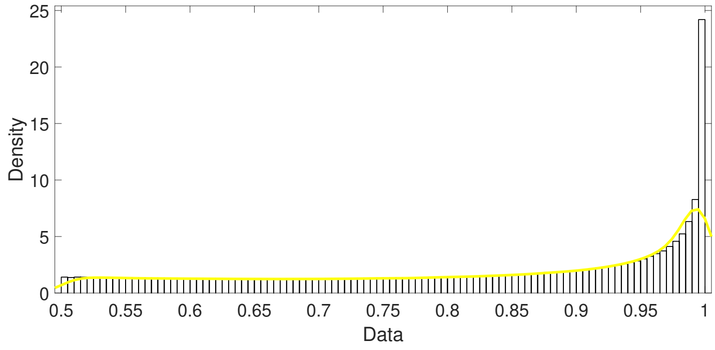

(d) Lung Opacity

Fig 5: **Fit of the non-parametric empirical distribution to the variance-covariance matrix data:** Class 0 (normal, blue dots), Class 1 (COVID-19, red dots), Class 2 (viral pneumonia, purple dots), and Class 3 (lung opacity, yellow dots).

Reviewer: *Remark 2.6)*
The density curves in Figure 5 do not show any statistical variances in the fit curves for Normal, COVID-19, and LungOp. Is this to be expected? Is this indicative of anything? How does the lack of variance in the curves affect the tuning weight parameter ω?

Authors: We appreciate the reviewer's insightful question. While the curves appear very similar, they exhibit subtle variations that may not be visually perceptible. These variations are confirmed by the smoothing parameter h statistics in the non-parametric distribution (see Table 2 below and Table 3 in the revised manuscript). Although minimal, these differences are sufficient for the ensemble model to capture class-specific characteristics. The parameter ω amplifies these variations, enhancing the model's ability to learn distinctive patterns for each class.
We added the following paragraph to the revised manuscript in line 372:

For illustration, Fig 5 depicts the non-parametric distribution fitted to the variance-covariance matrix data. While the curves corresponding to the four classes appear very similar, they exhibit subtle variations that may not be visually perceptible. These variations are confirmed by the statistics of the smoothing parameter h (see Table 2). Although minimal, these differences are sufficient for the ensemble model to capture class-specific characteristics. The parameter ω amplifies these variations, enhancing the model's ability to learn distinctive patterns for each class.

Table 2: **Statistics of the smoothing parameter h across all images and classes.**

	**Normal**	**COVID-19**	**Viral Pneumonia**	**Lung Opacity**
**mean±std**	0.061 ± 0.071	0.061 ± 0.070	0.061 ± 0.044	0.067 ± 0.092

Reviewer: *Remark 2.7)*
The wording of Line 378-379 does not make sense, what is the author trying to say.

Authors: We thank the reviewer for pointing out this ambiguity. The paragraph has been rewritten (lines 420 to 428 in the revised manuscript). We include the changes here for quicker assessment:

The tuning weight ω was found equal to $\{0.0142; 0.0173; 0.0143; 0.0161\}$, respectively for the normal, COVID-19, viral pneumonia, and lung opacity classes. The singular values ζ_k and the conditional indices η_k were found in the ranges $[0.003, 0.267]$ and $[0.061, 1.208]$, respectively. The rescaling mapped the features onto a different scale, where the classes are better discriminated. This rescaling significantly improved the performance of the ensemble learning classifier. S4 and S5 Figs, in the "Supporting Information" section, show the confusion matrices for training and testing, respectively. These results are aligned with the observed class separation shown in Fig 6(d).

Reviewer: *Remark 2.8)*
Figure 8 and Figure 9 are near identical and when overlaid on top of each other do not show any meaningful differences. Table 2 shows statistical differences between the singular and conditional values. I would therefore expect a difference in the predicted label scores for each parameter. Explain why the plots for the predicted table scores for the singular and conditional data should be near exactly the same or update plots to show how the data is different

Authors: We thank the reviewer for this critical remark. We apologize for the lack of clarity. Figures 8 and 9 are related to the "Partial dependence analysis" that we performed to validate the behaviour of the classification model.
May we refer the reviewer to our detailed response below to Question 3.4 by reviewer 3? We have added a new section in the revised manuscript explaining "Partial dependence analysis." We also included a section in the "Results and Discussion" explaining the results shown in Figures 8 and 9. We also adjusted the plots and captions of these two images.

Reviewer: *Remark 2.9)*
I would suggest updating order of datasets and legends to be consistent through out all figures. The order of the data in the figures change throughout the paper depending on the figure. Suggest using a consistent order in all figures, i.e. Normal, COVID-19, Viral Pneumonia, and Lung Opacity, so that reader doesn't have to read caption to determine order of data in each figure.

Authors: We thank the reviewer for their suggestion. According to the suggested order, we have now consistently ordered the classes throughout the manuscript.
Class 0 (normal), Class 1 (COVID-19), Class 2 (viral pneumonia), and Class 3 (lung opacity).

Reviewer: *Remark 2.10)*
In Figure 12, spell out the acronyms for ROC and AUC

Authors: We apologize for the confusion caused by the acronyms. We have now spelled them out in S3 Fig (which corresponds to Figure 12 in the initial version).
Receiver Operating Characteristic (ROC) and Area Under the Curve (AUC) of the features without the tuning weight.

RESPONSE TO REVIEWER #3

Reviewer: *This paper introduces a novel algorithm that leverages features derived from singular value decomposition (SVD) for image texture analysis, explicitly targeting the detection of abnormal X-ray images based on tissue attenuation. While the idea of using SVD for feature extraction has been explored in the past, the authors address the critical challenge of constructing meaningful and effective features from the decomposition. They propose using singular values and conditional indices as texture features, combined with a newly introduced tuning-weight parameter. This parameter, estimated through*

the coefficient of variation of the minimum covariance determinant from the variance-decomposition proportions of the SVD, accounts for the variability in tissue attenuation affected by pathologies.
The paper validates the proposed methodology using a challenging chest X-ray dataset with imbalanced classes, including COVID-19, viral pneumonia, lung opacity, and normal cases. The use of an ensemble bagged trees multiclass classification method achieved impressive accuracy rates: 88% without the tuning weight and 99% with it. The authors convincingly show that the proposed features, when enhanced by the tuning weight, improve key performance metrics such as True Positive Rate, False Negative Rate, Positive Predictive Value, False Discovery Rate, Area Under the Curve, Accuracy Rate, and Total Cost. Figures 6 and 7 clearly highlight the robustness of their approach.

The algorithms are well-documented, and the experimental results are thoroughly analyzed. The results are interesting and the paper is well-written. Only minor points require the authors' attention, if feasible.

Reviewer: *Remark 3.1)*
For readers who are not familiar with singular value decomposition (SVD) and principal component analysis (PCA), the authors should provide a brief explanation of how PCA approximates the original features. Specifically, it would be helpful to clarify the relationship between the original features and their lower-dimensional representation using PCA.

Authors: We thank the reviewer for their suggestion to add clarity to the paper. We have added the following paragraph, starting line 24, in the revised manuscript to define PCA and SVD and give a simple explanation of how SVD is used to calculate PCA.

In high-dimensional data analysis, such as image processing or computer vision, features can be correlated, making learning difficult. Various dimensionality reduction techniques exist, including subspace, manifold-based, and shallow and deep neural network methods. Principal Component Analysis (PCA) is a dimensionality reduction technique that belongs to subspace-based methods [1]. PCA aims to project the data onto fewer dimensions while preserving its inherent statistical patterns. Technically, PCA identifies the components along which the data matrix has the maximum variance. It has applications in data exploration, noise reduction, feature extraction, and data compression.

The most straightforward method to calculate PCA is through the conventional eigenvalue decomposition of the covariance matrix. However, Singular Value Decomposition (SVD) is the standard method used to prevent computational and numerical issues. SVD is a matrix factorization technique that decomposes a complex matrix X into the product of three matrices $X = U\Sigma V^T$, where U and V are orthogonal matrices containing the left and right singular vectors, respectively, and Σ is a diagonal matrix with singular values indicating the importance of each component. Principal components are extracted by selecting the desired number (k) of singular values and the corresponding columns of V (V_k). The lower-dimensional representation of X can then be computed $X' = XV_k$. For more details about PCA and SVD, we refer the reader to [2–4].

Reviewer: *Remark 3.2)*
The paper mentions that decision-making is based on identifying near dependencies between the columns of the image matrix X. This naturally raises the question of whether

analyzing the near dependencies between the rows (by transposing X and applying the same analysis) might yield additional insights. Have the authors explored this approach? If so, what were the results?

Authors: We appreciate the reviewer's insightful suggestion. We have not run the same analysis on rows in this study, as the study of columns provided interesting results. However, we share the reviewer's opinion on the potential of performing the same analysis on the rows by transposing the image. In addition, it can be interesting to combine both column and row analysis for analysing complex patterns in images. Features extracted from both dimensions can be used jointly to learn such complex patterns. We will certainly investigate this approach in our future work on appropriate data.

Reviewer: *Remark 3.3)*
In Figure 4, the term "tuning width" is mentioned but not clearly explained. What does this process involve, and how does it contribute to the proposed methodology?

Authors: The term "width" is a typo, and we sincerely apologize for the confusion. The correct term is **"tuning weight"** and has been corrected in the revised manuscript. This is the parameter ω that we derive using the coefficient of variation from the minimum covariance determinant of variance-decomposition proportions. It is used as a weight to multiply the conditional indices and the singular values to consider the variation of X-ray attenuation due to pathologies. The paper shows experimentally that this parameter effectively improves the differentiation of patterns corresponding to different pathologies. We have also modified the names of the various steps of our method in Figure 3 to improve readability.

Reviewer: *Remark 3.4)*
The x-axis labels in Figures 8 and 9 are unclear. Since these figures play a crucial role in showing the robustness of the proposed approach, the authors should provide more detailed explanations. In addition to the statement, "This feature combination suggests that these predictors are a powerful tool for discriminating between different classes of chest X-ray images," please elaborate on the specific judgments or insights that can be drawn from these figures.

Authors: We thank the reviewer for this remark. A new section (2.2.8 Partial dependence analysis) has been added to the methodology to explain the principle of "Partial dependency analysis". Please note that partial dependence analysis is a technique used in machine learning to interpret the relationship between a single feature (or a subset of features) and the model's prediction. It helps to understand how a given feature (or a subset of features) influences the predictions of the model while averaging out the effects of the other features. It consists of creating and interpreting partial dependence plots, like the ones in Figures 8 and 9. The technique is used for understanding feature importance and how they contribute to predictions. It is also important to validate model behaviour in healthcare applications of machine learning. Here we have used this technique to characterise the behaviour of the model depending on the variations of the two features.

We also updated the x-axis labels of Figures 8 and 9, and we included additional explanations.

Partial dependence analysis

To analyse the behaviour of the classification model and assess the effect of the two features on the predictions, we performed the partial dependence analysis. This technique is used in machine learning to interpret the relationship between a single feature (or a subset of features) and the model's prediction. It helps to understand how a given feature (or a subset of features) influences the predictions of the model while averaging out the effects of the other features. It consists of creating and interpreting partial dependence plots, like the ones in Figures 8 and 9. The technique is utilised to understand feature importance and how they contribute to predictions. It is also essential to validate the behaviour of models in healthcare applications.

Let $f(\Theta)$ denote a predictive model, with the feature vector $\Theta = (\Theta_1, \Theta_2, ..., \Theta_p)$. For binary classification, $f(\Theta)$ is the probability estimate $P(y = 1|x)$. Let Θ_S denote a small subset of Θ, with its complement $\Theta_C = \Theta \setminus \Theta_S = \{\theta_{C,1}, ..., \theta_{C,N_C}\}$, of size N_C. The partial dependence between Θ_S and the model's output is defined as:

$$\hat{f}_S(\Theta_S) = \mathbb{E}_{\Theta_C}[f(\Theta_S, \Theta_C)], \tag{1}$$

where $\mathbb{E}_{\Theta_C}$ is the expectation taken over the marginal distribution of X_C in the dataset. In practice, given the discrete nature of the data, $\hat{f}_S(\Theta_S)$ is computed as the average over all values of Θ_C:

$$\bar{f}_S(\Theta_s) = \frac{1}{N_C} \sum_{i=1}^{N_C} f(\Theta_S, \theta_{C,i}). \tag{2}$$

For K classes, the generalized partial dependence for class k is:

$$\hat{f}_k(\Theta_S) = \mathbb{E}_{\Theta_C}[P(y = k|\Theta_S, \Theta_C]. \tag{3}$$

Equation (Eq.3) means that for each class k, the probability is marginalized over the complementary features, with the aim of analyzing how $P(y = k)$ varies with Θ_S. For a comprehensive treatment of partial dependence analysis, we refer the reader to [4].

In our study, $\Theta = (\Theta_1, \Theta_2) = (\omega\zeta_k,\ \omega\eta_k)$ and $K = 4$. We calculated and displayed the partial dependence plots for each class, to assess the effect of singular values and conditional indices on the model's decision. We performed this analysis with and without applying the tuning weight.

Results and discussion

We also included the following explanations in Section "Results and discussion" (section 3, line 429) in the revised manuscript, to explain the partial dependence plots depicted in figures 8 and 9.

Figs 8 and 9 show the partial dependence plots of the two features, without and with the tuning weight. The curves show the effect of the features on predicting each class

separately. Figs 8(a) and 8(b) show the compared effect of the tuning weight with the conditional indices on the model's behaviour. We can observe that the model has similar behaviour for conditional indices between 0 and 20 and their corresponding weighted values. In this range, the model favours the class "lung opacity" (till index 8), then has more confidence in predicting "viral pneumonia" (between 8 and 18), and then switches to predicting "COVID-19" with high confidence (index around 18). However, after applying the tuning weight, the effect is obvious: the model predicts "COVID-19" with high confidence and high probability, starting from the weighted value of 0.02. This observation aligns with our results, showing that the classification model with weighted features performs significantly better.

Similarly, when comparing Figs 9(a) and 9(b), the model behaves similarly with and without the tuning weight for singular values between 0 and 4. The model first favours "COVID-19" in this range with significant confidence. Then, it becomes more confident in predicting the "normal chest" class with significant probability. However, with the weighted singular values, the model predicts the "normal chest" class with high confidence and probability.

Using the jointly weighted conditional indices and the weighted singular values, it appears clear that the model finds a combined pattern that allows it to differentiate the four classes and, more importantly, "COVID-19" from "normal chest".

Considering all the plots from Figs 8 and 9, it is reasonable to hypothesise that using the four features together will be useful for learning complex image patterns. However, we did not test this hypothesis in this study, as the results were good with only the weighted features.

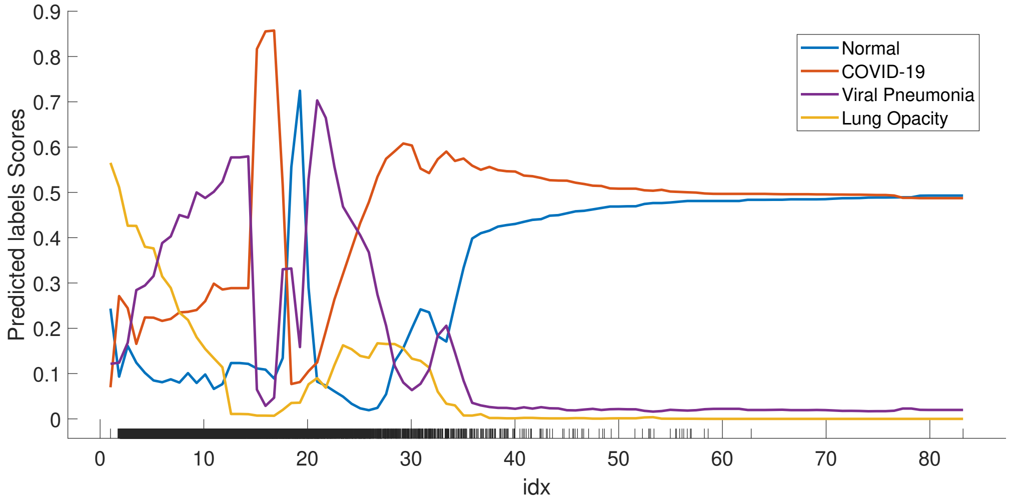

(a) Conditional indices without ω

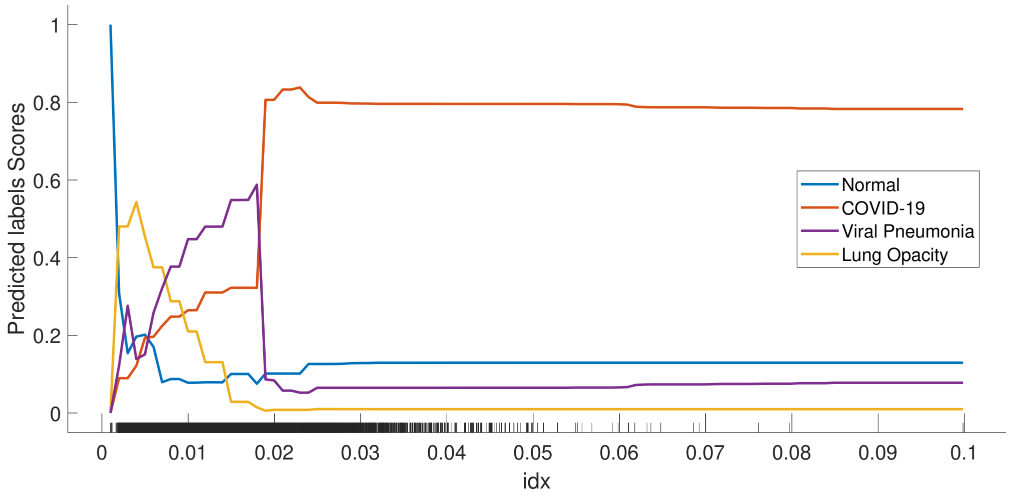

(b) Conditional indices with ω

Figure 8: **Partial dependency plots of conditional indices during testing, with and without the tuning weight ω:** Class 0 (normal, blue dots), Class 1 (COVID-19, red dots), Class 2 (viral pneumonia, purple dots), and Class 3 (lung opacity, yellow dots). The x-axis values are the conditional indices in (a) and the weighted conditional indices in (b). Values of the y-axis are probabilities of predicting a class. For example, in (a), the probability of predicting COVID-19 is 0.9 when the conditional index is around 18. Curves show the variation of the probabilities of predicting each class depending on the values of the feature. Figure (b) shows that for values of the weighted conditional indices starting from 0.2, the model is highly confident in predicting COVID-19, with a high probability (more than 0.8).

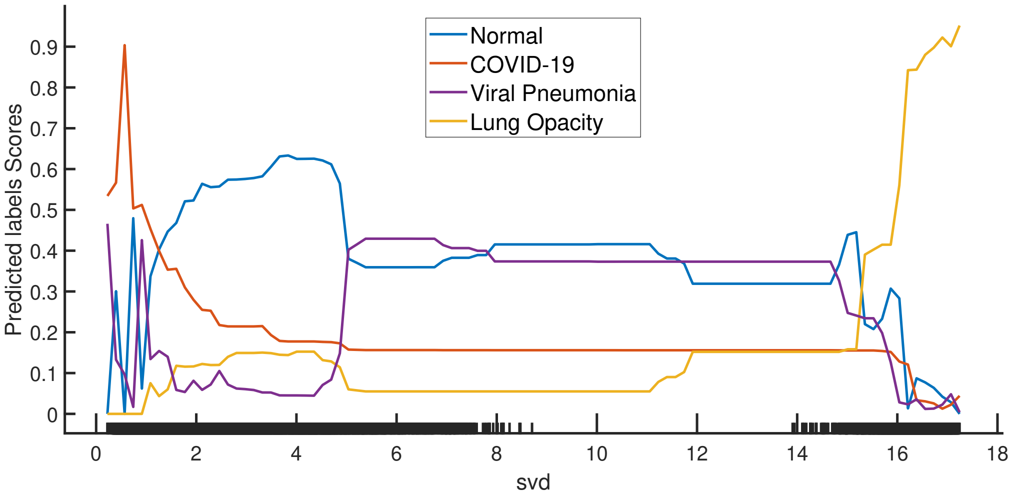

(a) Singular values without ω

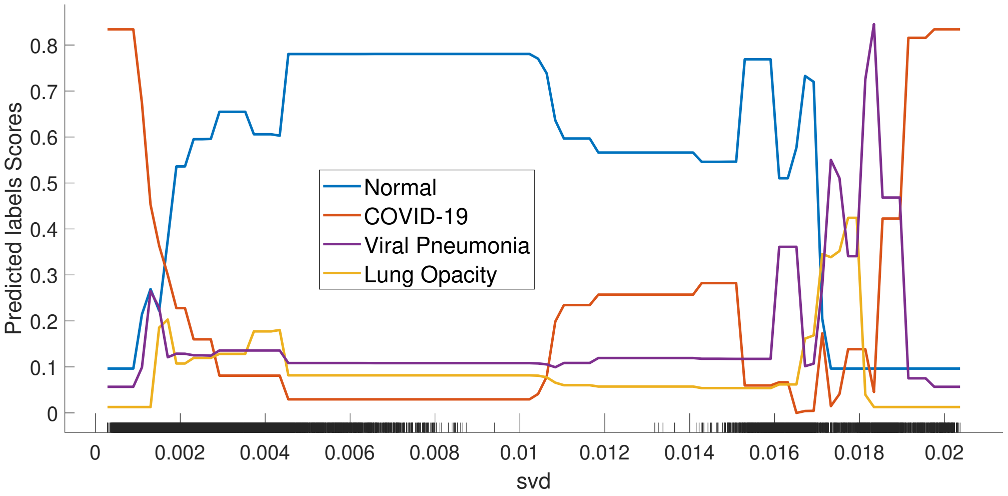

(b) Singular values with ω

Figure 9: **Partial dependency plots of singular values during testing, with and without the tuning weight ω:** Class 0 (normal, blue dots), class 1 (COVID-19, red dots), class 2 (viral pneumonia, purple dots), and class 3 (lung opacity, yellow dots). The x-axis values are singular values in (a) and weighted singular values in (b). Values of the y-axis are probabilities of predicting a class. For example, in (a), the probability of predicting COVID-19 is 0.9 when the singular value is around 0.40. Curves show the variation of the probabilities of predicting each class depending on the values of the feature. Comparing plots in (a) and (b), we notice that the behaviour of the model is similar in the range $[0, 5]$. However, Figure (b) shows that for the weighted singular values between $[0.005, 0.010]$, the model is highly confident in predicting normal chest, with high probability (close to 0.8).

Reviewer: *Remark 3.5)*
In the conclusion, the paper claims, "In addition to its excellent performance, the proposed

method has a low computational cost with imbalanced data, compared to other methods." However, this statement is difficult to agree with, given that performing SVD is computationally expensive, especially for large matrices. Although low-rank approximations of SVD can reduce costs, the paper does not clarify whether this was used. If the proposed method is indeed computationally efficient, please specify the baseline or methods to which it is being compared. Most of the conventional feature extraction methods listed in Table 1 are not computationally expensive, so this comparison needs further clarification.

Authors: We agree with the reviewer and we acknowledge that our statement about the low computational cost of our method may have been overstated. The statement of lower cost was motivated by the comparison with current deep-learning methods. In reality, we do understand that the main component of the computational cost of our method is related to the computation of the SVD decomposition. In our experiments, we used the augmented Lanczos bidiagonalization algorithm, through the svds function in Matlab. We calculated only the 6 first singular values, leading to relatively fast computation time on a standard PC computer. To avoid ambiguity, we have added the following paragraph at the end of the section on SVD (Section 2.2.1, line 220).

Computing the SVD decomposition is the main computational cost component in the proposed method. When applied to large dimension $(M \times N)$ images, direct SVD decomposition is costly given its $\mathcal{O}(MN \min(M, N))$ complexity. In this work, we used the augmented Lanczos bidiagonalization algorithm [5], implemented in the function *svds* in MATLAB®. This algorithm computes the first C singular values with a complexity of $\mathcal{O}(MNC)$, hence reducing the complexity.

We also down-stated our claim in the conclusion in line 486 by replacing "low cost" with "a descent computational cost with imbalanced data, compared to current deep learning methods."

Reviewer: *Remark 3.6)*
Typos

- *quatify: in the first sentence of section 2.2 Methodology*
- *there are several places where 'comma' is missing before 'where'*
- *inconsistency in placing a space before 'where'*

Authors: We thank the reviewer for pointing out these typos. In the revised manuscript, we have corrected all these typos and used appropriate punctuation.

References

[1] Van Der Maaten L, Postma EO, Herik VD. Dimensionality reduction: A comparative review. Technical Report TiCC TR 2009-005. Tilburg centre for Creative Computing, Tilburg University; 2009.

[2] Jolliffe IT. Principal Component Analysis. Springer; 2002.

[3] Yanai H, Takeuchi K, Takane Y. Projection matrices, generalized inverse matrices, and singular value decomposition. Springer; 2011.

[4] Hastie T, Tibshirani R, Friedman J. The Elements of Statistical Learning: Data Mining, Inference, and Prediction. Springer Nature; 2017.

[5] Baglama J, Reichel L. Augmented Implicitly Restarted Lanczos Bidiagonalization Methods. SIAM Journal on Scientific Computing. 2005;27(1):19–42. doi:10.1137/04060593X.
